# Salivary Glucose Oxidase from Caterpillars Mediates the Induction of Rapid and Delayed-Induced Defenses in the Tomato Plant

**DOI:** 10.1371/journal.pone.0036168

**Published:** 2012-04-30

**Authors:** Donglan Tian, Michelle Peiffer, Erica Shoemaker, John Tooker, Eric Haubruge, Frederic Francis, Dawn S. Luthe, Gary W. Felton

**Affiliations:** 1 Department of Entomology, Center for Chemical Ecology, Penn State University, University Park, Pennsylvania, United States of America; 2 Functional and Evolutionary Entomology, Gembloux Agro-Bio Tech, University of Liege, Liege, Belgium; 3 Department of Crop and Soil Science, Center for Chemical Ecology, Penn State University, University Park, Pennsylvania, United States of America; Max Planck Institute for Chemical Ecology, Germany

## Abstract

Caterpillars produce oral secretions that may serve as cues to elicit plant defenses, but in other cases these secretions have been shown to suppress plant defenses. Ongoing work in our laboratory has focused on the salivary secretions of the tomato fruitworm, *Helicoverpa zea*. In previous studies we have shown that saliva and its principal component glucose oxidase acts as an effector by suppressing defenses in tobacco. In this current study, we report that saliva elicits a burst of jasmonic acid (JA) and the induction of late responding defense genes such as proteinase inhibitor 2 (*Pin2*). Transcripts encoding early response genes associated with the JA pathway were not affected by saliva. We also observed a delayed response to saliva with increased densities of Type VI glandular trichomes in newly emerged leaves. Proteomic analysis of saliva revealed glucose oxidase (GOX) was the most abundant protein identified and we confirmed that it plays a primary role in the induction of defenses in tomato. These results suggest that the recognition of GOX in tomato may represent a case for effector-triggered immunity. Examination of saliva from other caterpillar species indicates that saliva from the noctuids *Spodoptera exigua* and *Heliothis virescens* also induced *Pin2* transcripts.

## Introduction

The ability of many plant species to mount induced defenses to herbivores has been documented in what is becoming a voluminous literature [1],[2]. A plethora of inducible direct and indirect chemical defenses along with inducible physical defenses are known [3],[4],[5]. Perhaps one of the best studied plant systems with regards to induced responses to wounding and/or herbivory is the tomato plant *Solanum lycopersicum* ( = *Lycopersicon esculentum*) [6],[7],[8],[9],[10],[11].

An “antinutritional” cocktail of proteins is induced in response to caterpillar feeding in S.*lycopersicum* including polyphenol oxidases [12],[13],[14]; various proteinase inhibitors [15],[16]; ascorbate oxidase [17]; leucine aminopeptidase [6]; arginase [11] and threonine deaminase [11]. The expression of many of these proteins is regulated by the octadecanoid pathway [18],[19],[20]. In addition to these proteins, the role of glandular trichomes in resistance to insect feeding is being explored [21]. Glandular type VI trichomes in tomato are a formidable defense against some herbivores. The production of these trichomes is also dependent upon jasmonic acid signaling [22],[23]. The glandular trichomes can be induced by wounding or application of methyl jasmonate [24].

The use of mutants, deficient in the phenotypic expression of induced systemic defenses, led to the conclusion that herbivore defense signaling in tomato is largely dependent upon the octadecanoid pathway [25],[26]. The peptide hormone systemin acts upstream from jasmonic acid and likely promotes long-distance defense responses by amplifying jasmonate production in vascular tissues as a first line of defense signaling [8],[27]. Adding further complexity to the defense signaling network is the finding that in addition to systemin, several other hydroxyproline-rich glycopeptides associated with the cell wall matrix act as defense signals [28],[29]. The octadecanoid pathway is part of a complex signaling network that can be positively or negatively regulated by signal cross talk from other hormones and messengers [30]; these include salicylic acid [31],[32],[33], nitric oxide [34], abscisic acid [35], ethylene [36], auxin [37], brassinosteroids [38], and hydrogen peroxide (H_2_O_2_) [39],[40].

Because of the complexity of the signaling networks, there are multiple points at which caterpillar secretions may intercept or amplify signaling components. Noctuid caterpillars such as *Helicoverpa zea* (*H. zea*) produce during feeding a chemically rich complex of secretions from the labial salivary glands. We have previously shown that glucose oxidase from the labial glands may suppress wound-induced accumulation of nicotine in tobacco [41]. The mandibular glands are comparatively small in *H. zea* and are poorly characterized, but we have identified the protein glucose oxidase as well as several carotenoids as components [42],[43]. Regurgitant is known to contain scores of proteins [44], as well as fatty acid-glutamine conjugates such as volicitin, which elicit the production of plant volatiles–important components of indirect defense and plant-plant signaling [45],[46].

Based upon our previous studies in tobacco that demonstrated that salivary glucose oxidase was an effector suppressing induced defenses [47], we initiated this investigation in tomato to examine the role of caterpillar secretions in mediating defense gene expression and the production of glandular trichomes.

## Results

### Quantification of Caterpillar Secretions and GOX Activity

On average we can collect about 0.5 nl of saliva from each *H. zea* caterpillar. Although the volume is small, the saliva collected from 10 caterpillars contains about 560 ng of protein, as measured by a modified Bradford assay [48]. In comparison, we can collect about 5 µl regurgitant from each caterpillar and the regurgitant from 10 caterpillars contains about 6 µg of protein. Due to these very small volumes, we have chosen to measure GOX activity after combining saliva with 30 µl PBS. GOX activity assays showed that only *H. zea* saliva had high activity (see below), while the regurgitant had very low, nearly undetectable GOX activity.

### Proteomic Analysis of *H. zea* Saliva

**Figure 1:**
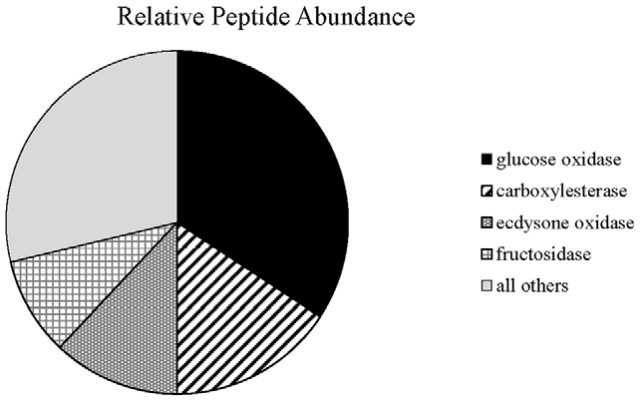
Relative abundance of peptides identified by proteomic analysis of *H. zea* saliva.

We performed shotgun proteomic analysis of secreted salivary proteins from *H. zea* to identify potential protein candidates for plant defense gene elicitation. The number of mass spectral counts obtained for each protein provides a quantitative measure of protein abundance [49]. Of the 33 proteins that were identified (Table S1), glucose oxidase (GOX) was by far the most abundant protein accounting for 34% of the identified proteins (Fig. 1). Carboxylesterase, ecdysone oxidase, and fructosidase were the next most abundant proteins. These results are in general agreement with the proteomic analysis of saliva from the closely related species *Helicoverpa armigera* [50]. GOX is exceptionally active in *H. zea* saliva with ca. 17 μmol/min/mg protein activity. This is more than 7X greater specific activity than what we reported in labial salivary glands [51]. Fructosidase activity was detectable, but we could not detect carboxylesterase or ecdysone oxidase activity in secreted saliva using customary substrates. Because one of the reaction products during the hydrolysis of sucrose by fructosidase is D-glucose, the enzyme may complement the action of GOX by providing additional substrate for GOX activity.

### Effects of Caterpillar Secretions on *Pin2* Defense Gene Expression

**Figure 2:**
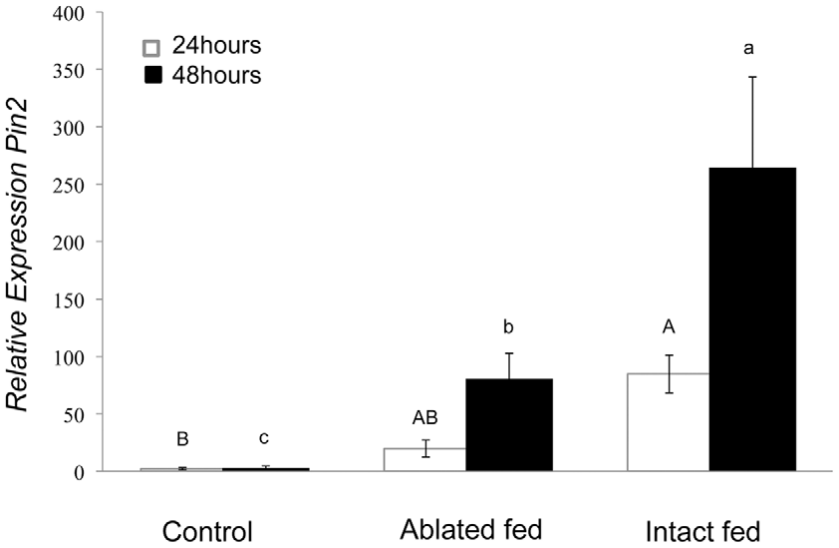
Relative expression of *Pin2* in MicroTom leaves 24 and 48 **h after** ***H.zea*** **feeding (Fisher's P<0.05 following ANOVA).** Error bars represent mean ±SE.

*Pin2* was chosen as a defense marker gene because it has been well characterized as a defense in tomato [52],[53]. Feeding by *H. zea* larvae significantly induced *Pin2* expression in MicroTom tomato leaves (Fig. 2); after 24 h feeding, the *Pin2* expression was significantly higher than untreated control plants (ANOVA, F_(2,6)_ = 17.46, P = 0.03). After 24 h of feeding, however, there was no significant difference in *Pin2* expression induced between damage caused by caterpillars whose spinneret was intact and individuals with ablated spinnerets, which renders them unable to release saliva [50]. But after 48 h, feeding by intact caterpillars significantly induced greater *Pin2* expression than feeding by ablated caterpillars (F_(2,6)_ = 36.25, P<0.001). To verify that the ablation procedure successfully prevented release of saliva, we performed a tissue blot of leaves fed on by the treated caterpillars [54]. We used an antibody to detect GOX protein and found that ablation strongly inhibited the release of the salivary proteins (Fig. S1).

**Figure 3:**
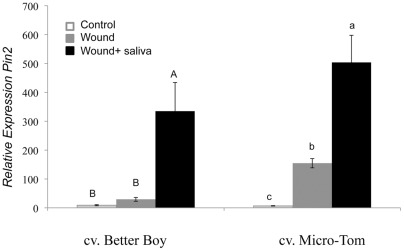
Relative expression of *Pin2* in Better Boy and MicroTom leaves 48 h after wounding and application of *H. zea* saliva (Fisher's P<0.05 following ANOVA). Error bars represent mean ±SE.

These data show that *H. zea* saliva plays an important role in induction of *Pin2*. In order to further confirm the role of saliva in inducing defense gene expression, we wounded and applied saliva to Better Boy and MicroTom leaves. The amount of salivary protein (∼0.5 μg) used in these experiments is a very conservative estimate of how much is secreted during feeding [54]. Using an antibody to detect salivary GOX, we estimated that larvae secreted>1.5 μg GOX during a 4 h period [54]. The application of *H. zea* saliva to wounded leaves significantly induced the expression of *Pin2* after 48 h in both Better Boy and MicroTom cultivars compared to the wounded control (ANOVA, Better Boy, F_(2,6)_ = 8.5, P = 0.007; MicroTom F _(2,6)_ = 70.09, P<0.001; Fig. 3). In contrast, we found that regurgitant did not significantly affect *Pin2* expression compared to the wounded PBS control treatment (ANOVA, (F_(3,8)_ = 2.71, P = 0.115) (Fig. S2).

### Saliva and Other Defense Genes

**Figure 4:**
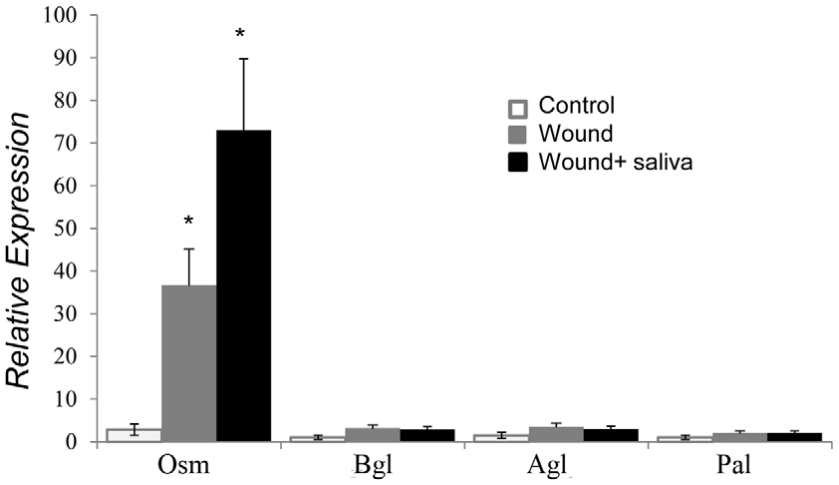
Relative expression of defense genes in Better Boy 24 h after wounding and application of *H. zea* saliva or PBS (Fisher's P<0.05 following ANOVA). Osm  =  osmotin; Agl  =  acidic glucanase; Bgl  =  basic glucanase; Pal  =  phenylalanine lyase. Error bars represent mean ±SE.

To determine if saliva is affecting other signaling pathways in the tomato plant, we applied saliva to wounded plants and measured the relative expression of four genes typically induced by pathogens. Leaves treated with saliva had significantly higher expression of osmotin gene, which resistance to bacterial pathogen [55], compared to untreated controls, but were not different than wounded leaves with PBS applied (Fig. 4, ANOVA, F_(2,6)_ = 6.85, P = 0.028). Acidic and basic glucanases and phenylalanine ammonia lyase (*Agl*, *Bgl* and *Pal*), which are induced by pathogens [56] were not induced in wounding or saliva treatments.

### Saliva and Early-Responding Signaling Genes

**Figure 5:**
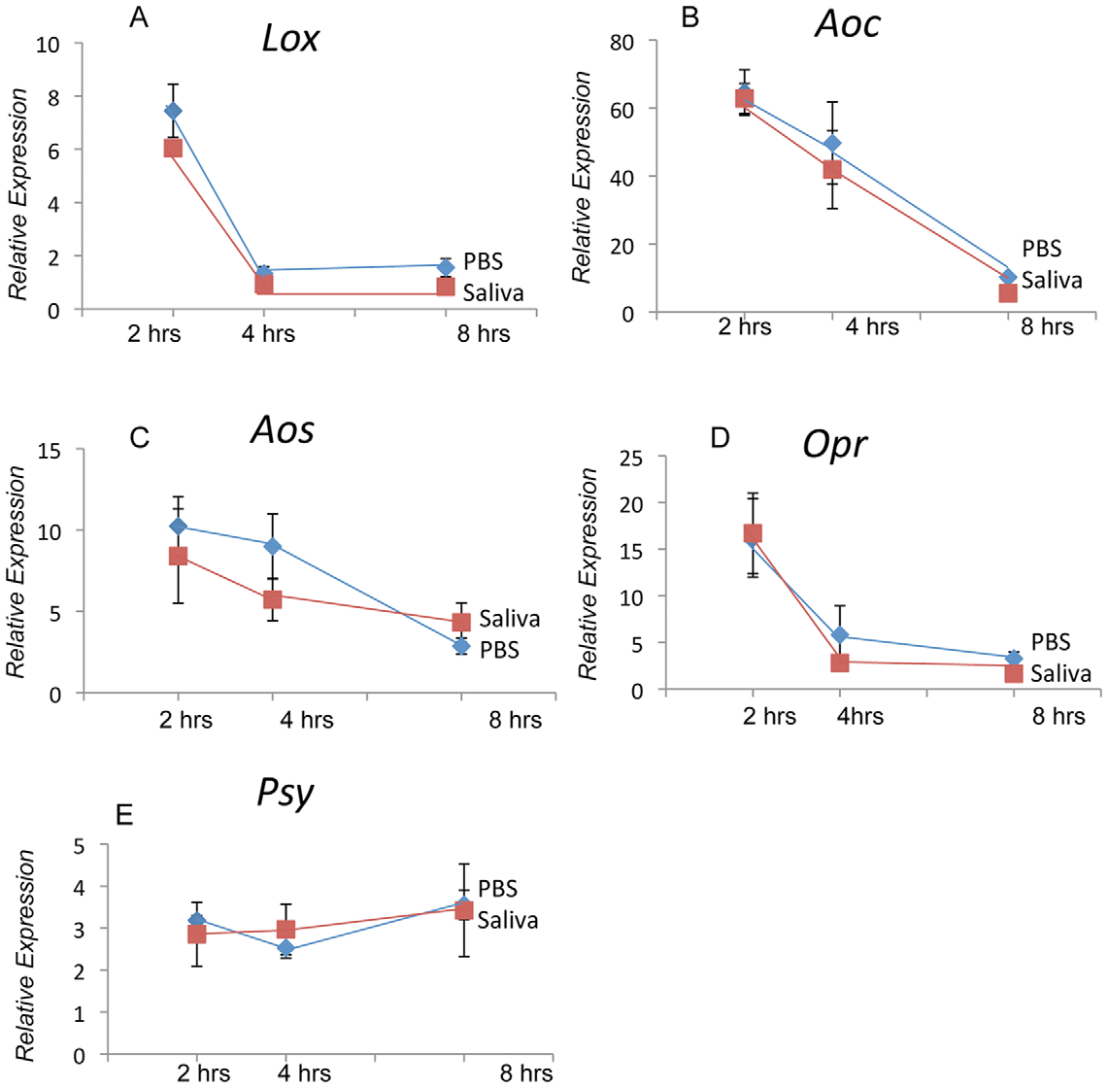
Relative expression of early responding genes in Better Boy 2, 4 and 8 **h after wounding and application of** ***H. zea*** **saliva or PBS (Fisher's P<0.05 following ANOVA).** **A:** Lipoxygenase D; **B:** Allene Oxide Cyclase; **C:** Allene Oxide Synthase; **D:** Osmotin; **E:** Prosystemin.

We examined the effects of saliva on “early responding” signaling genes associated with the octadecanoid pathway at several time points post wounding [40]. We found that saliva did not affect the expression of lipoxygenase D (*LoxD*), allene oxide synthase (*Aos*), allene oxide cyclase (*Aoc*), 12-oxophytodienoate reductase (*Opr3*), or prosystemin (*Psy*) at 2, 4, or 8 h post wounding (Fig. 5).

### Effect of Glucose Oxidase on Defense Gene Expression in Different Tomato Tissues

Because GOX was the most abundant salivary protein and earlier reports showed that infiltration of tomato petioles with a H_2_O_2_ generating system consisting of glucose and fungal glucose oxidase triggered *Pin2* expression [40], we tested the effect of GOX and saliva on *Pin2* expression in MicroTom tomato leaves, green and red fruit, and flower tissues. We applied fungal GOX at levels consistent with the amount secreted by caterpillars [54]. The fungal GOX has very similar substrate specificity as the GOX from *H. zea* salivary glands [51] and thus serves as appropriate model for the insect enzyme.

**Figure 6:**
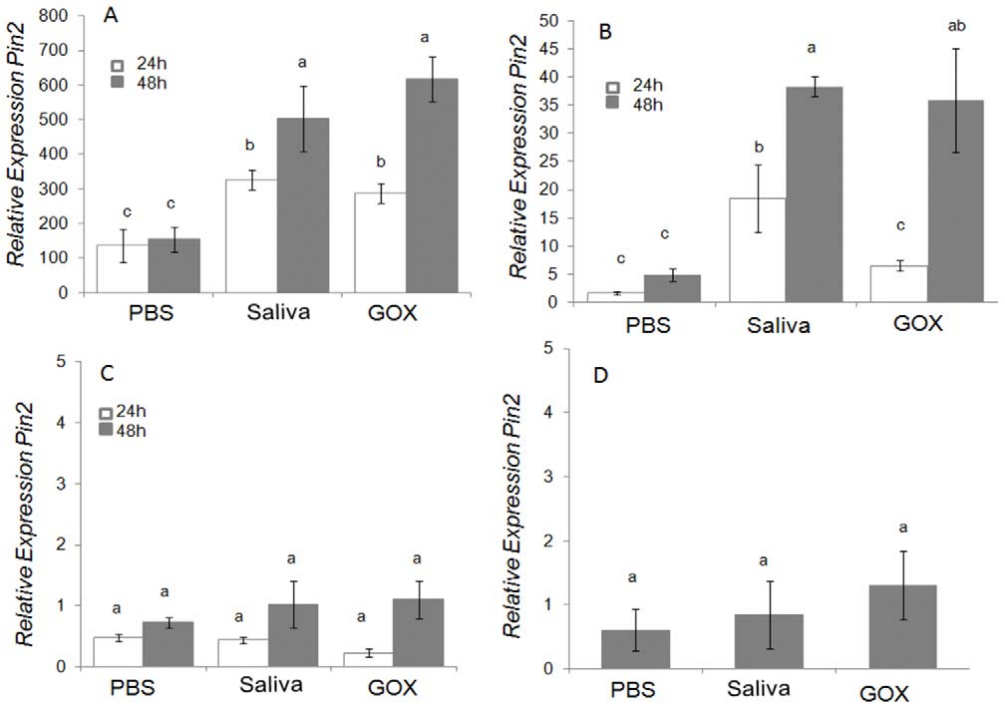
Relative expression of *Pin2* on different MicroTom tissues 24 and 48 **h after wounding and treatment application.** **A:** Wounded leaf; **B:** Green fruit; **C:** red fruit, **D:** Flower receptacle. Error bars represent ±SE.

Relative expression of the late responding gene *Pin2* was examined at two time points after wounding and application of PBS, *H. zea* saliva or fungal GOX on MicroTom leaves, green and red fruit, and flowers (Fig. 6). Wounding with PBS, saliva or GOX both induce *Pin2* expression on the leaf at 24 and 48 h. However the *H. zea* saliva and GOX treatment significantly induced more *Pin2* expression than PBS treatment at both time points. At 48 h, the *Pin2* expression in leaf treatment with saliva and GOX is significantly higher than at 24 h time points, while the PBS treatments at the different times were not significantly different. The effect of induction of *Pin2* expression by GOX and saliva was not significantly different at the two time points (Fig. 6A, ANOVA, F_(5,12)_ = 20.70, P<0.001). Treatments of green fruit with wounding and PBS, saliva or GOX showed *Pin2* induction relative to the unwounded control. Compared to PBS, the saliva and fungal GOX treatments induced *Pin2* expression in green fruit after wounding. At 24 h, the saliva treatment induced higher *Pin2* than the GOX treatment, while at 48 h; there were no significant differences between saliva and GOX (Fig. 6B, ANOVA, F_(5,12)_ = 14.39, P<0.001). But with red mature fruit, wounding with PBS, saliva or GOX treatment caused no significant *Pin2* induction compared with non-treated fruits (Fig. 6C, ANOVA, F_(5,12)_ = 4.43, P = 0.058). Floral tissues had higher constitutive *Pin2* expression level, but again there was no significant response to wounding or saliva in the flower tissues (Fig. 6D, ANOVA, F_(2,6)_ = 0.28, P = 0.761).

### Effect of Saliva on Plant Hormones

**Figure 7:**
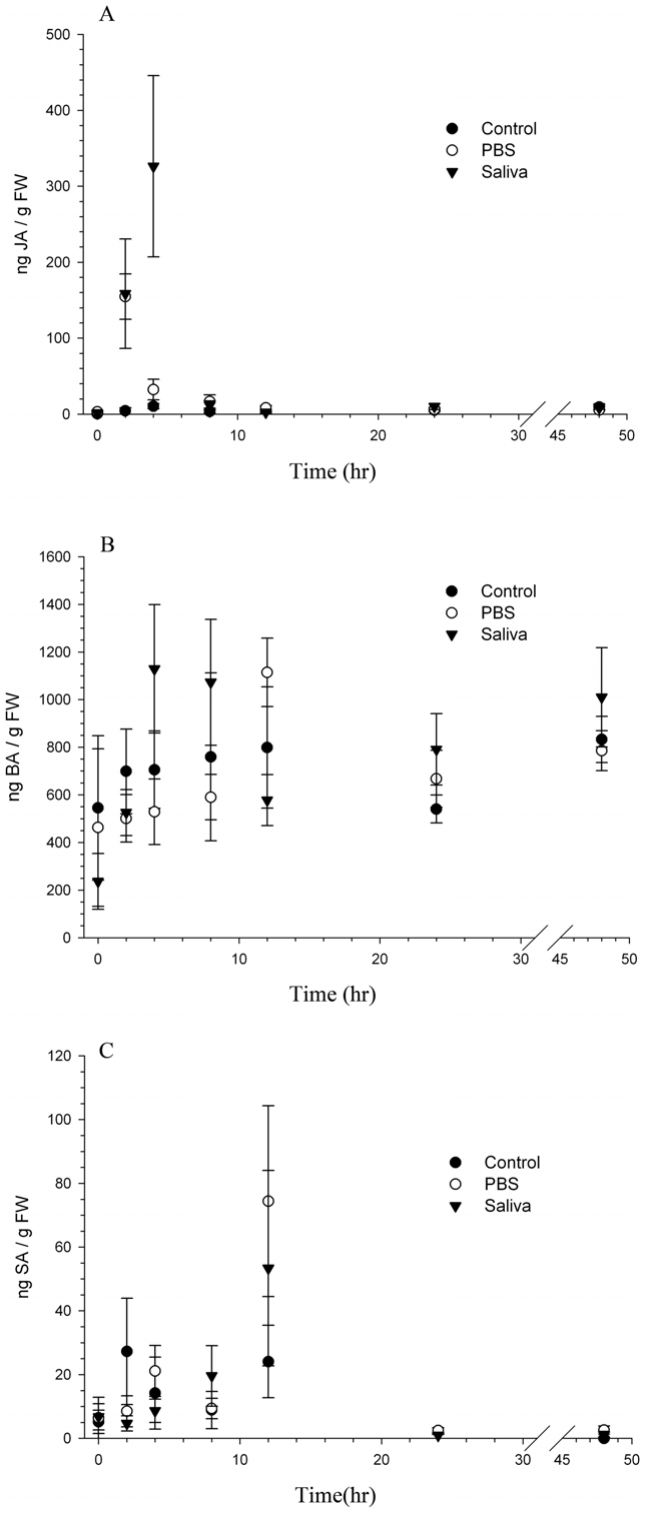
Levels of plant hormones in leaves of undamaged plants and wounded plants that received either control buffer (PBS) or *H. zea* saliva in buffer. **A:.** Jasmonic Acid. Asterisks indicate significant differences in JA levels between the three treatments (Tukey's HSD P<0.005 following ANOVA on log-transformed data; each time point was independent of the others and was analyzed as such; α = 0.007 following a Bonferroni adjustment; data are shown untransformed); **B:** Benzoic acid; **C:** Salicylic acid.

We examined the effect of saliva on several defense-associated plant hormones including jasmonic acid, salicylic acid, and benzoic acid, a precursor to salicylic acid. Samples collected immediately after applying the PBS or saliva treatments showed that JA levels were equal across plants (Fig. 7; ANOVA F_(2,14)_ = 1.5, P = 0.27). After two hours, however, JA levels in wounded plants receiving PBS or *H. zea* saliva were significantly elevated above levels in undamaged control plants (Fig. 7; ANOVA F_(2, 14)_ = 31.7, P<0.0001). After four hours, JA levels of PBS-treated plants had dropped and equaled those in control plants, but levels of JA in saliva-treated plants remained significantly elevated and were ∼13 and 40 times greater than those measured in PBS-treated and control plants, respectively (Fig. 7. ANOVA F_(2,14)_ = 15.5, P = 0.0004). For the remaining four sampling periods (8, 12, 24, and 48 h), all three treatments had similar JA levels (Fig. 7; ANOVA F_(2, 14)_<0.82, P>0.45).

Saliva had no significant effects on salicylic or benzoic acid levels during the time period examined (Fig. 7; SA: ANOVA F_(2,14)_<2.4, P>0.13; BA: ANOVA F_(2,14)_<2.8, P>0.10).

### Trichome Induction by *H. zea* Saliva

**Figure 8:**
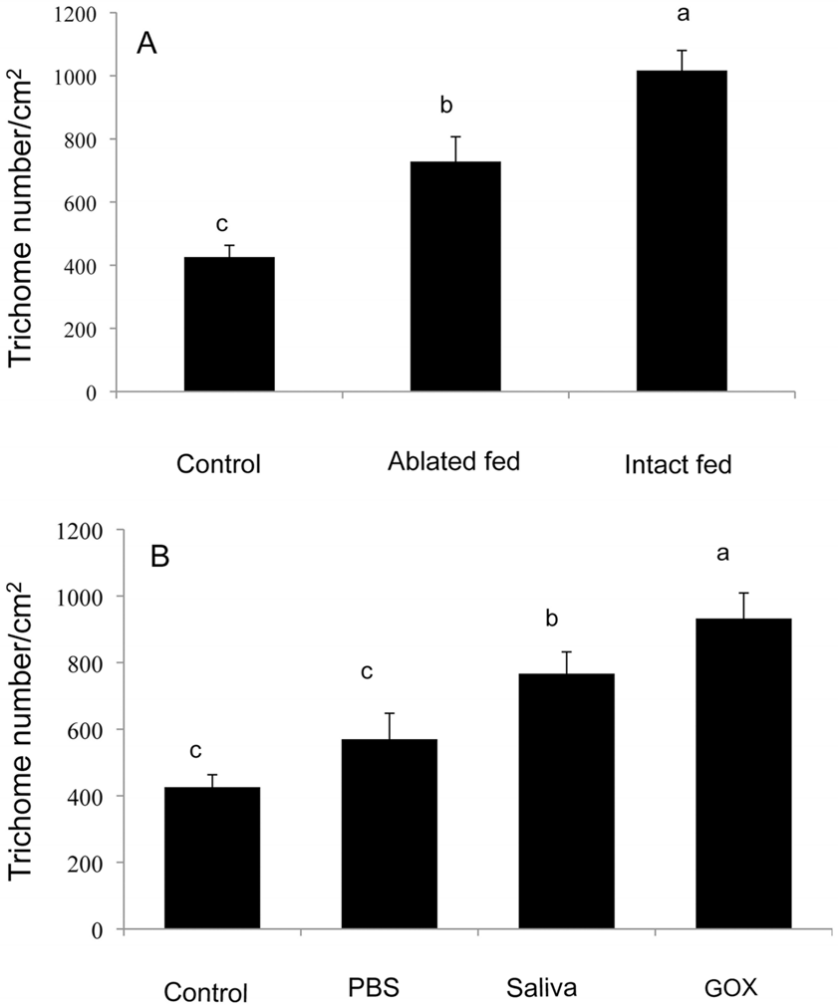
Average number of trichomes on MicroTom leaf disc 2 weeks after wounding or insect feeding. A. Ablated and Non-ablated insect feeding; **B.** Wounding and treatment with *H. zea* saliva or fungal glucose oxidase (2 ng/µl), non-wounding and wounding with PBS buffer treatment as control. Data shown represent mean ±SE.

When the number of type VI glandular trichomes on MicroTom leaves was counted two weeks after *H. zea* feeding, plants fed on by caterpillars with intact spinnerets had a significantly higher density of trichomes than plants attacked by caterpillars with ablated spinnerets, which in turn were significantly greater than unwounded control plants (Fig. 8A, ANOVA, F_(2,57)_ = 11.84, P = 0.012). These results indicate that secretion of saliva induces the production of type VI glandular trichomes in new leaves.

To further examine the effect of caterpillar saliva on trichome induction, plants were wounded and saliva or purified GOX was immediately applied to the wound site. Plants treated with saliva and GOX had significantly more trichomes than untreated or PBS treated control plants (Fig. 8B, ANOVA, F_(3,76)_ = 7.42, P<0.001).

### Effect of Saliva from other Caterpillar Species on Defense Gene Expression

**Figure 9:**
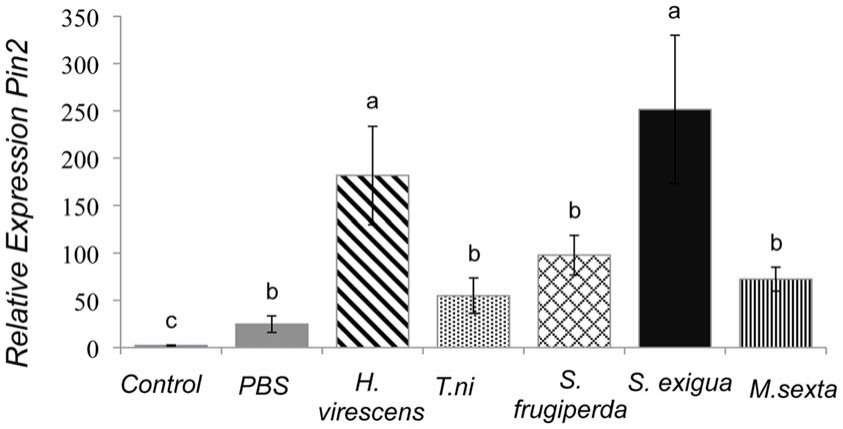
Relative expression of *Pin2* in Better Boy tomato leaves 48 **h after wounding and application of salivary gland homogenate from different caterpillar species.**

Because saliva could not be collected directly from the spinneret of the other caterpillar species, we prepared salivary gland homogenates and applied 20 μg proteins per wound as previously described. Salivary homogenates from *Trichoplusia ni* (*T. ni*), *Manduca sexta* (*M. sexta*), and *Spodoptera frugiperda* (*S. frugiperda*) did not elicit *Pin2* expression at levels higher than the PBS wounded control (Fig. 9). Salivary homogenates from *Heliothis virescens* (*H. virescens*) and *Spodoptera exigua* (*S. exigua*) induced significantly greater *Pin2* transcript levels than the wounded control (P<0.05).

## Discussion

Salivary glucose oxidase is found in aphids [57] and many caterpillar species [50],[58] and may function as an effector to suppress defenses in multiple plant species including *Nicotiana tabacum* [41],[59], *Nicotiana attenuata* [60], *Medicago truncatula* [61], and *Arabidopsis thaliana* [62]. One of the enzymatic products of the GOX reaction, H_2_O_2_, is believed to be the main factor responsible for the suppression [41],[59]. Indeed H_2_O_2_ is a major regulator of plant gene expression [63] and plays a key role in defense signaling against plant pathogens [64]. In *N. attenuata*, GOX and associated H_2_O_2_ elicit a salicylic acid (SA) burst, but decrease the jasmonate and ethylene levels [60]. It is widely known that SA antagonizes JA responses [32]. Thus the mechanism of action of GOX in these systems is antagonism of the JA pathway via elicitation of SA. This is analogous to the production of the JA mimic, coronatine, by certain strains of *Pseudomonas syringae* bacteria; coronatine effectively suppresses the SA-pathway via cross-talk [65],[66].

In marked contrast in tomato, treatment of plant tissue with the H_2_O_2_-generating enzyme fungal GOX plus glucose resulted in the induction of JA-regulated defensive genes (>4 h) such as proteinase inhibitor (*Pin2*) and not the early-expressed JA signaling-related genes [40]. Because SA antagonizes *Pin2* expression in tomato [31], presumably the GOX system did not elicit SA production as seen in other plant species such as tobacco. Furthermore, antisense NADPH-oxidase tomato plants have reduced levels of H_2_O_2_ and a diminished expression of late wound-response genes [67]. In our experiments the application of saliva elicited a significant, but transient burst in JA while having no impact on SA levels (Fig. 7). This response is different than that observed in other plant systems and accounts for different action of GOX on induced defenses in tomato. Consistent with the findings published earlier [40], GOX did not impact expression of early signaling genes (i.e., *LoxD*, *Opr3*, *Aos*, *Aoc*), but did elicit higher levels of the late-responding gene *Pin2*.

We observed that treatment with salivary gland homogenates from various species could also induce *Pin2* expression. These differences could, in part, be explained by the GOX levels in the salivary glands of the caterpillar species which follow the pattern of highest to lowest activity: *H. zea* > *H. virescens* > *S. frugiperda* >*S. exigua* >*T. ni* >*M. sexta* [57]. The species with the lowest GOX activity, *M. sexta* and *T. ni*, elicited the weakest response in *Pin2*. We provide a cautionary note with these data because salivary gland homogenates may contain scores of proteins that are not secreted and thus could confound the outcome or interpretation of the experiments.

In addition to induction of JA and JA-regulated transcripts, we observed that saliva and GOX induced delayed defenses, namely the induction of glandular trichomes. Glandular trichomes in tomato are regulated by the jasmonate pathway [23],[24] and it is not surprising that we found that wounding and herbivory caused an induction of trichomes in newly formed leaves. There have been earlier reports in several other plant species that herbivory may cause induction of trichomes [68],[69],[70],[71],[72]. However, salivary factors may be responsible for the observations that feeding in black mustard by different herbivores (i.e., *Pieris rapae* cabbage looper, *Trichoplusia ni*, and the mustard flea beetle, *Phyllotreta cruciferae*) elicit differential effects on induction of leaf trichome density [68]. The authors did not compare the salivary GOX activity in these species, but our findings indicate that at least one known salivary factor, GOX, triggers trichome production in tomato. This provides the first evidence that salivary factors are an additional factor responsible for regulating the induction of trichomes. Because this is a delayed-induced defense, it is likely that the increased trichome densities will impact subsequent generations of herbivores attacking the plant. Furthermore, induction of trichomes in the maternal generation results in greater trichome density in the offspring [73].

The role of GOX as an effector has been noted in the studies cited earlier. In the case of tomato, GOX appears to induce JA-regulated defenses above and beyond the levels observed by wounding or feeding alone. In the plant pathology literature it is noted that successful pathogens produce effectors to suppress plant immunity, but in turn, plants have evolved additional receptors (i.e., R-proteins) to perceive the pathogen effectors and thus mount an additional defense called “effector-triggered immunity” [74],[75]. Our results here indicate that tomato can recognize a component of herbivore offense andt this could be analogous to R genes evolving to recognize phytopathogenic effectors, but further investigation is needed to identify the receptor(s) responsible for perceiving GOX and its enzymatic products. Overall there is a lack of understanding of not only effectors, but also herbivore associated molecular patterns (HAMPs) that requires elucidation of receptors responsible for recognition and the specific signaling pathways involved with their recognition [76],[77].

Noctuid herbivores produce multiple secretions during feeding that have the potential to mediate the induction of direct and indirect plant defenses [78]. In one example with the tobacco budworm *Heliothis virescens*, the effect of the combination of saliva and oral secretions or regurgitant on induced volatiles was examined [79]. In this case it was found that saliva had an inhibitory effect on volatile induction and that both saliva and regurgitant were necessary to elicit the “volatile signature” of *H. virescens* feeding. The results in our current paper using the closely related *H. zea* indicate that secretions from the labial salivary glands and not regurgitant are involved with the induction of direct defenses in the tomato plant. Regurgitant from *H. zea*, at the amounts tested, did not affect the expression of the defense gene *Pin2*. Although the exact amounts of OS that are secreted by larvae are not known, we used amounts that are consistent with what has been published in the literature for many lepidopteran species [80],[81],[82]. Regurgitant from the tobacco hornworm *Manduca sexta* elicits *Pin2* expression in tomato [83] and the related species *Solanum tuberosum* [84]. The regurgitant of *M. sexta* does contain the fatty acid conjugates (FACs) elicitors [85],[86],[87],[88], but a different blend than what occurs in *H. zea* [45]. Furthermore, the effects of FACs from caterpillars are host-plant specific, where in some cases they do not play a role in mediating indirect or direct defenses [89]. In another herbivore species of tomato, regurgitant from the Colorado potato beetle *Leptinotarsa decemlineata*, suppressed the induction of both *Pin1* and *Pin2* transcripts [83],[90]. The fact that regurgitant from *H. zea* had no significant effect on the JA-regulated defenses argues that saliva is the primary secretion mediating induced defenses in tomato to this insect. The ultimate outcome of the interactions of herbivores with their host plant will depend upon the relative balance of herbivore associated molecular patterns (HAMPs) and effectors produced by the herbivore and the ability of the plant to perceive these chemical cues and respond appropriately.

## Materials and Methods

### Insects and Plant Material

Lepidopteran eggs (*H. zea, H. virescens*, and *M.sexta*) were obtained from the insectary at North Carolina State University. *S. frugiperda* were obtained from USDA-ARS, Mississippi State, MS. *Spodoptera exigua* and *T. ni* were provided by Jim Tumlinson, Penn State University. All species were reared as previously described [91]. Tomato seeds of cv. Better Boy and MicroTom were obtained commercially (Tomato Growers Supply, P.O. Box 60015, Fort Myers, FL 33906), grown as previously described [54] and used at the 4 node stage. For all induction experiments, plants were maintained in the greenhouse under 800 W Super Spectrum lights (Sunlight Supply Co., Vancouver, WA) with a 16∶8 light dark cycle.

### Saliva and Regurgitant Collection

To collect *H. zea* saliva, 5^th^ instar larva were chilled on ice. Flaccid larva were then immobilized in a metal hair clip and observed with a dissecting microscope. As the larva returned to room temperature, the secretion from the spinneret was collected via capillary action into a gel loading pipet tip (VWR, West Chester, PA) containing glycerol. Saliva in glycerol was stored at −80°C until needed. Regurgitant was collected directly from the oral cavity of 5^th^ instar larva and stored at −80°C. The GOX activity was determined according to the method described [51]. Briefly, glucose, peroxidase, and o-dianisidine were used in the reaction cocktail with spectrophotometric detection at 460 nm.

Salivary glands (i.e., labial glands) of the caterpillars (*H. virescens*, *T. ni*, *S. exigua*, *S. frugiperda*, and *M. sexta*) were dissected and homogenized as previously described [51].

### Proteomics of *H. zea* Saliva

For proteomic identification of saliva proteins, saliva was collected as described above, except 5 mM EDTA in 50 mM Tris-HCl, pH 8.0 was used in place of glycerol. Saliva was collected from 100 *H. zea* larva into 30 µl of buffer and stored at −80°C. NanoLC was carried out using a Dionex Ultimate 3000 (Milford, MA). Mobile phase solvents A and B were 0.1% TFA (v/v) in water and 0.1% TFA (v/v) in 80% Acetonitrile. Tryptic peptides were fractionated at a flow rate of 0.6 μl/min using linear gradients and the following program: 5% B for 5min, 5 to 15% B over 5 min, 15 to 60% B over 40 min, 60 to 95% B over 1 min and hold for 5 min. The mobile phase was ramped back to the initial conditions. Fractions were collected at 20-seconds intervals followed by Mass Spectrometry analysis on Applied Biosystems Proteomics Analyzer. Peptides were searched against the Insecta database (taxid:50557). Proteins with Total Ion C.I.% greater than 95 are considered high confidence matches.

### Effect of Insect Feeding on Induction of *Pin2*

*H. zea* were reared and ablated as previously described [54]. After ablation the larva were allowed to recover and feed on artificial diet for 7 h, then starved overnight. Larvae were caged individually with a small clip cage on the fourth leaf for 6 h and then removed. The control treatment consisted of an empty cage tomato plants. Each treatment replicated three times for each time point. Wounded leaves were harvested 24 and 48 h after caterpillar feeding, frozen in liquid nitrogen, and stored at −80°C for later RNA extraction.

### Wounding with Saliva Application and Induction of *Pin2*

To test how *H. zea* saliva affects *Pin2* induction in leaves, we wounded the youngest fully expanded leaf and applied saliva. Two holes, each 3 mm diameter, were punched in the midvein of the terminal leaflet. Immediately, the saliva collected from 10 caterpillars and combined with 20 µl PBS was applied to the wound site. PBS treated plants were wounded in the same way and had 20 µl PBS added; control plants were unwounded without any treatment. For regurgitant wound treatment, 20 µl collected regurgitant was applied at wounded site. Three replicates were used for all treatments. Treated leaves were harvested 24 h and 48 h after treatment, frozen in liquid nitrogen, and stored at −80°C for later RNA extraction.

To test if saliva induces defense genes in tomato fruit and flower stages, we used MicroTom tomatoes. Green fruit was tested two weeks after flowering and red fruit when they begin to turn red. Fruits were wounded by punching one hole at the side of the fruits. MicroTom flowers were wounded at the pedicel. For fruits and flowers, immediately after wounding, 20 μl of PBS buffer, *H. zea* saliva combined with 20 µl PBS, or fungal GOX (2 ng/µl) combined with PBS was pipetted onto the edges of the wound. Control plants were unwounded for leaf, flower or fruit tissues. Three replicates were used for all the treatments. After 24 or 48 hours, wounded tissue was harvested and frozen in liquid nitrogen for RNA and real-time PCR.

### Quantitative Real Time PCR

Tissue (100 mg), harvested from the area around the wound, was homogenized in liquid nitrogen and total RNA extracted with RNeasy Plus Mini-kit (Qiagen, Valencia, CA). 1 μg purified RNA was used with High Capacity cDNA Reverse Transcription kit (Applied Biosystems, Foster City, CA ) to create cDNA. Real-time PCR primers were designed using Primer Quest Software (Applied Biosystems) (Table S2). All reactions used Power SYBR Green PCR Master Mix and were run on a 7500 Fast Real-Time PCR System (Applied Biosystems).

The housekeeping gene ubiquitin was used to normalize C(T) values [92]. Relative quantifications, with unwounded plants as the reference group, were then calculated using the 2^-▵▵C(T)^ method [93]. To validate this analysis method, primer efficiency was analyzed by comparing the normalized C(T) values of 5 serial dilutions of cDNA.

#### Phytohormone Analysis

To measure JA, SA, and BA in wounded leaves, plants were wounded and saliva or PBS applied as described above. One hundred mg of leaf tissue was then collected into FastPrep^®^ tubes (Qbiogene, Carlsbad, CA) containing 1 g of Zirmil beads (1.1 mm; Saint-Gobain ZirPro, Mountainside, NJ), and frozen at −80°C until processing. We used five independent replicates per time point and per treatment. To extract and detect JA, SA, and BA, we used a previously described method which was slightly modified [94], [95]. Briefly, we derivatized the carboxylic acids to methyl esters, which were isolated using vapor phase extraction and analyzed by GC-MS with isobutane chemical ionization using selected-ion monitoring. Our method deviated from that of the previous authors in that we quantified amounts of methyl jasmonate (meJA), methyl salicylate (meSA), and methyl benzoate (meBA) using standard curves made with the pure compound (Sigma-Aldrich, St. Louis, MO), relying on internal standards to confirm derivatization and recovery. To confirm the identity of meJA, meSA, and meBA in our samples, we analyzed extracts by GC-MS with electron ionization, comparing retention times and spectra with that of the pure compound.

### Trichome Induction

To learn how *H. zea* secretions affect glandular trichome induction, intact and ablated *H. zea* were allowed to feed on MicroTom tomato leaves as described above. In a second experiment MicroTom leaves were wounded and treated with PBS, saliva, or fungal GOX as described above. After treatment, plants were maintained in the greenhouse for two weeks then type VI trichomes were counted on the new growth leaves as previously described [24]. Twenty plants were treated for each treatment.

### Effect of Saliva from Other Caterpillar Species on Defense Gene Expression

The homogenized glands were diluted and adjusted with PBS to obtain 20 μg of protein in 20 μl of homogenate. Plants were wounded and treated with the homogenate as previously described. The experiment was replicated at three separate times with seven replicates per treatment. *Pin2* expression was assayed at 48 h post-wounding.

### Statistical Analysis

Data were analyzed using general linear models for analysis of variance (ANOVA) where appropriate with post-hoc comparison of means using the Fisher-LSD means separation (Mintab Software, State College, PA).

For statistical analysis, relative expression values were analyzed by ANOVA (Minitab Inc., State College, PA) using Fisher's separation of means (P<0.05).

## Supporting Information

Figure S1 **Tissue blot of** ***H. zea*** **saliva secreted during feeding on MicroTom leaves** **.** Ablated and intact H. zea were allowed to feed on detached leaves, then proteins on the leaf were electro-blotted onto nitrocellulose. GOX antibody was used to detect glucose oxidase on the leaf and visualized with Vector ABC kit and DAB substrate.
(TIF)

Figure S2 **Relative expression of** ***Pin2*** **in tomato leaves 24** **h and 48** **h after wounding and application of** ***H. zea*** **regurgitant.** Effect of regurgitant is not significantly different than PBS (F = 2.71, P = 0.115). Error bars represent ±SE.
(TIF)

Table S1 **Proteomic Identification of Salivary Proteins in** ***H. zea*** **.**
(DOCX)

Table S2 **Primers used for real-time PCR assays of relative expression.**
(DOCX)
