# Supplementary figures and images for: Salivary Glucose Oxidase from Caterpillars Mediates the Induction of Rapid and Delayed-Induced Defenses in the Tomato Plant
Source: PLoS One. 2012 Apr 30;7(4):e36168. doi: 10.1371/journal.pone.0036168 (PMC3340365; doi:10.1371/journal.pone.0036168)

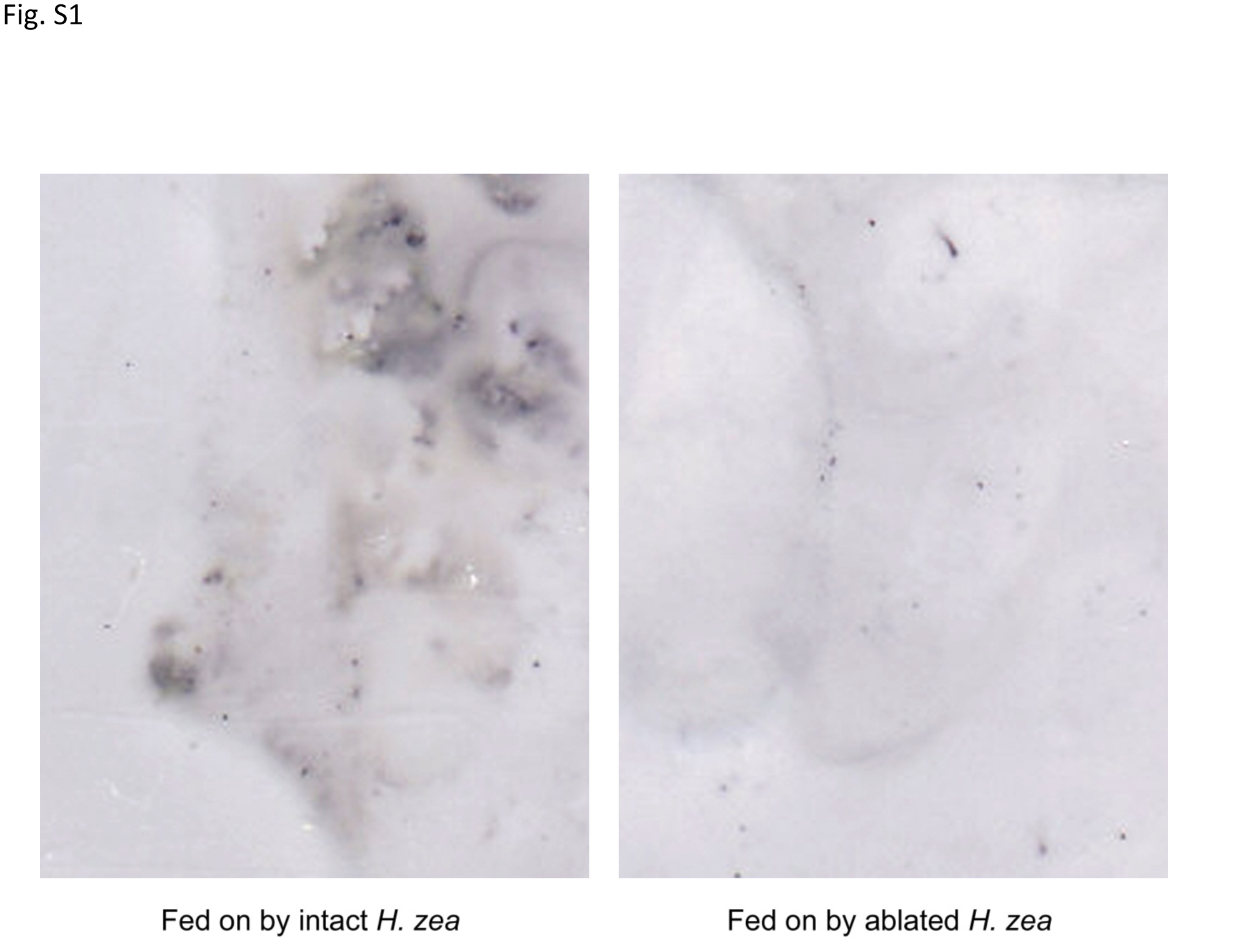

Supplement: Figure S1 — Tissue blot of H. zea saliva secreted during feeding on MicroTom leaves . Ablated and intact H. zea were allowed to feed on detached leaves, then proteins on the leaf were electro-blotted onto nitrocellulose. GOX antibody was used to detect glucose oxidase on the leaf and visualized with Vector ABC kit and DAB substrate. (TIF) [file pone.0036168.s001.tif]

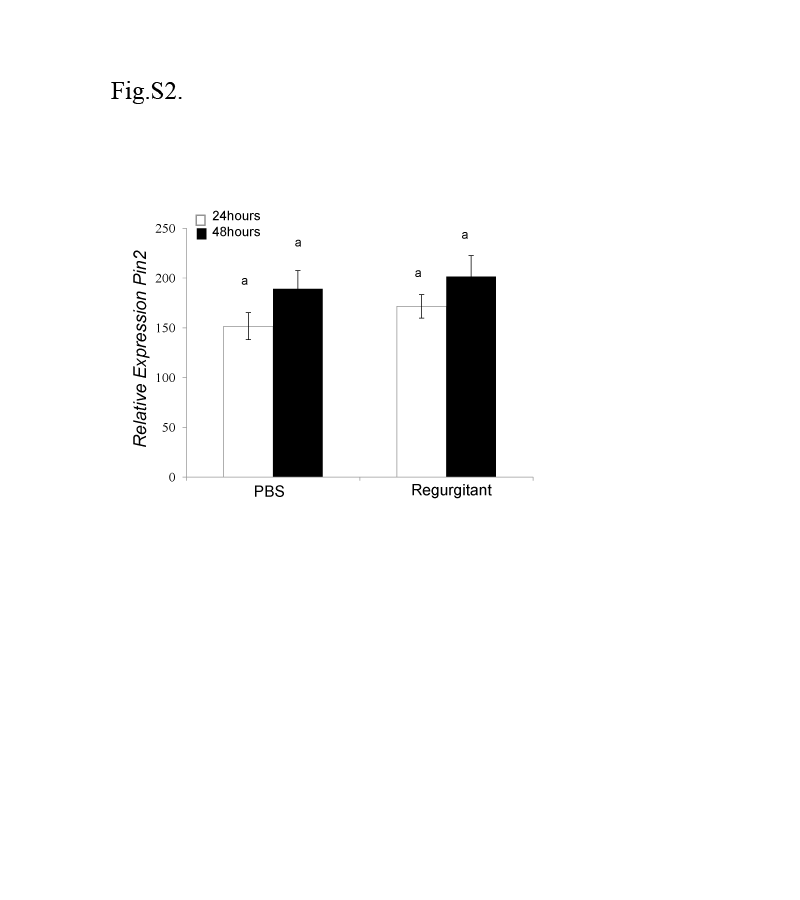

Supplement: Figure S2 — Relative expression of Pin2 in tomato leaves 24 h and 48 h after wounding and application of H. zea regurgitant. Effect of regurgitant is not significantly different than PBS (F = 2.71, P = 0.115). Error bars represent ±SE. (TIF) [file pone.0036168.s002.tif]
