# Supplementary material for: Salivary Glucose Oxidase from Caterpillars Mediates the Induction of Rapid and Delayed-Induced Defenses in the Tomato Plant
Source: PLoS One. 2012 Apr 30;7(4):e36168. doi: 10.1371/journal.pone.0036168 (PMC3340365; doi:10.1371/journal.pone.0036168)
Supplement: Table S1 — Proteomic Identification of Salivary Proteins in H. zea . (DOCX) [file pone.0036168.s003.docx]

Table S1. Proteomic Identification of Salivary Proteins in *Helicoverpa zea*

|  | Protein identification | Organism | NCBI Accession | # of Peptides | Total Ion Score | MW | pI |
| --- | --- | --- | --- | --- | --- | --- | --- |
| 1 | glucose oxidase | *Helicoverpa zea* | gi\|215982092 | 20 | 1658 | 66939.1 | 5.3 |
| 2 | glucose oxidase-like enzyme | *Helicoverpa armigera* | gi\|186909546 | 17 | 1373 | 66858.9 | 5.0 |
| 3 | carboxyl/choline esterase CCE016d | *Helicoverpa armigera* | gi\|294846818 | 13 | 1243 | 61508.4 | 4.8 |
| 4 | putative ecdysone oxidase | *Helicoverpa zea* | gi\|219815604 | 10 | 680 | 63822.7 | 5.4 |
| 5 | fructosidase | *Helicoverpa armigera* | gi\|156968287 | 3 | 316 | 53971.8 | 4.9 |
| 6 | epoxide hydrolase | *Trichoplusia ni* | gi\|2661096 | 1 | 116 | 52813.6 | 8.6 |
| 7 | GF21896 | *Drosophila ananassae* | gi\|194765569 | 1 | 86 | 65895.2 | 6.1 |
| 8 | aryl-alcohol oxidase precursor, putative | *Ixodes scapularis* | gi\|241592310 | 1 | 64 | 62991.9 | 6.7 |
| 9 | GM16912 | *Drosophila sechellia* | gi\|195356860 | 1 | 62 | 19478.5 | 10.4 |
| 10 | AGAP001021-PA | *Anopheles gambiae str. PEST* | gi\|58376848 | 1 | 61 | 112289.2 | 6.7 |
| 11 | GH18582 | *Drosophila grimshawi* | gi\|195036744 | 1 | 58 | 35803.1 | 7.2 |
| 12 | GH18646 | *Drosophila grimshawi* | gi\|195036490 | 1 | 58 | 205225.0 | 8.9 |
| 13 | PREDICTED: similar to alaserpin, partial | *Acyrthosiphon pisum* | gi\|193629771 | 1 | 57 | 39086.8 | 5.4 |
| 14 | wd-repeat protein | *Aedes aegypti* | gi\|157133770 | 1 | 56 | 54519.2 | 4.4 |
| 15 | hypothetical protein TcasGA2_TC013848 | *Tribolium castaneum* | gi\|270007291 | 1 | 56 | 36914.2 | 9.7 |
| 16 | esterase | *Plutella xylostella* | gi\|22324345 | 1 | 56 | 11776.8 | 4.7 |
| 17 | GJ14811 | *Drosophila virilis* | gi\|195401845 | 2 | 55 | 320874.0 | 6.5 |
| 18 | GL10752 | *Drosophila persimilis* | gi\|195150013 | 1 | 53 | 59935.6 | 5.4 |
| 19 | dachshund | *Culex quinquefasciatus* | gi\|170037058 | 1 | 53 | 67174.6 | 6.6 |
| 20 | PREDICTED: similar to nuclear receptor subfamily 2, group E, member 3 | *Acyrthosiphon pisum* | gi\|193683726 | 1 | 53 | 54684.5 | 8.7 |
| 21 | serine protease | *Culex quinquefasciatus* | gi\|170036773 | 1 | 53 | 17642.0 | 9.1 |
| 22 | Putative aminopeptidase W07G4.4 | *Camponotus floridanus* | gi\|307185505 | 1 | 52 | 46591.9 | 6.8 |
| 23 | CG4593-PA | *Drosophila ananassae* | gi\|269972528 | 1 | 52 | 24610.3 | 5.8 |
| 24 | cytochrome P450 4C1 | *Culex quinquefasciatus* | gi\|170059524 | 1 | 52 | 29405.6 | 5.4 |
| 25 | GI18727 | *Drosophila mojavensis* | gi\|195123267 | 1 | 51 | 6764.8 | 3.8 |
| 26 | hypothetical protein AaeL_AAEL009299 | *Aedes aegypti* | gi\|157121612 | 1 | 51 | 75960.2 | 9.0 |
| 27 | hypothetical protein EAG_00548 | *Camponotus floridanus* | gi\|307166578 | 1 | 51 | 31249.9 | 9.2 |
| 28 | protein C9orf39, putative | *Pediculus humanus corporis* | gi\|242019086 | 1 | 50 | 140011.8 | 8.9 |
| 29 | carboxylesterase clade A, member 5 | *Nasonia vitripennis* | gi\|289177094 | 1 | 50 | 60470.7 | 5.8 |
| 30 | GF19006 | *Drosophila ananassae* | gi\|194770241 | 1 | 50 | 997115.9 | 5.9 |
| 31 | CG41265, isoform B | *Drosophila melanogaster* | gi\|161075927 | 1 | 49 | 95193.6 | 8.9 |
| 32 | CG33127 | *Drosophila melanogaster* | gi\|28573984 | 1 | 49 | 30895.5 | 5.1 |
| 33 | GF21209 | *Drosophila ananassae* | gi\|194763357 | 1 | 49 | 70846.1 | 7.1 |
