# Supplementary material for: Salivary Glucose Oxidase from Caterpillars Mediates the Induction of Rapid and Delayed-Induced Defenses in the Tomato Plant
Source: PLoS One. 2012 Apr 30;7(4):e36168. doi: 10.1371/journal.pone.0036168 (PMC3340365; doi:10.1371/journal.pone.0036168)
Supplement: Table S2 — Primers used for real-time PCR assays of relative expression. (DOCX) [file pone.0036168.s004.docx]

Table S2. Primers used for real-time PCR assays of relative expression

| Primer name | Gene Name/ Accession number | Primer sequence (5'-3", forward/reverse) |
| --- | --- | --- |
| *Agl* | Acidic beta (1,3) glucanase/ x74905 | AAT CGC GCT CCT TTG CTT GTC AAC / AAG GCC AAC CAC TCT CCG ATA CAA |
|  |  |  |
| *Aoc* | Allene Oxide Cyclase/aj308481 | AGC CTC TGC TGC TCT TAG AAC CAT / AGG GCT TCC ACG ATC ACG TTC ATT |
|  |  |  |
| *Aos* | Allene Oxide Synthase/af230371 | ACC CGT TTA GCA AAC GAG ATC CGA / CGT TGC AAA TGG TTG GTA CCC GAA |
|  |  |  |
| *Bgl* | Basic beta (1,3) glucanase/ x74906 | ATC AAC CAA CTC TCC AAG CCC TCA / GCA CAT ACT GCG CGT TGC CAT TTA |
|  |  |  |
| *Lox* | Lipoxygenase D/u37840 | GTT CAT GGC CGT GGT TGA CAC ATT / TGG TAA TAC ACC AGC ACC ACA CCT |
|  |  |  |
| *Osm* | Osmotin precursor/ AF093743 | AGT ACG CCT TGG ACC AGT TTA GCA / CAC ATG GAC CTT GGG TGC AAC AAT |
|  |  |  |
| *Pal* | Phenylalanine ammonia lyase/ M83314 | TTC GAG TTG CAG CCT AAG GAA GGA / ATA GCA GCA GCC TCA ATC TGA CCA |
|  |  |  |
| *Pin2* | Wound inducible proteinase inhibitor 2/ K03291 | GGA TTT AGC GGA CTT CCT TCT G / ATG CCA AGG CTT GTA CTA GAG AAT G |
|  |  |  |
| *Psy* | Prosytemin/m84801 | ACC AAA GGT GGA ACA TGA GGA AGG / TTG GGA GGA TCA CGC TTT GAT GGA |
|  |  |  |
| *Ubi* | Ubiquitin/ X58253 | GCC AAG ATC CAG GAC AAG GA / GCT GCT TTC CGG CGA AA |
